# Supplementary material for: Larvicidal Activities against Aedes aegypti of Supernatant and Pellet Fractions from Cultured Bacillus spp. Isolated from Amazonian Microenvironments
Source: Trop Med Infect Dis. 2021 Jun 17;6(2):104. doi: 10.3390/tropicalmed6020104 (PMC8293452; doi:10.3390/tropicalmed6020104)
Supplement: Supplementary file 1 [file tropicalmed-06-00104-s001.zip › tropicalmed-1236643-supplementary.pdf]

Supplementary Material

Table S1. *Bacillus* strains isolated from soil and water samples from the municipalities of Parintins, Manaus and Coari, Amazonas state, Brazil. Species assignment of every queried sequence would be the desired outcome, but in many cases the limited resolution of the 16S rRNA locus precluded an exact classification at the species level. Taxonomic classification at the genus level was then assigned to the isolated strains.

Strain	Culture medium / Substrat	Geographic origen	% Identity	Species GenBank acc. number
SX17	NA/ Water	Dona Chagas – Mao	100%	*Achromobacter xylosoxidans* MT052620
SPa05	NA/ Soil	Parananema - Pin	100%	*Achromobacter xylosoxidans* MT052658
Apr10	ISP2/ Water	Parananema - Pin	100%	*Bacillus* sp MT052598
SUF02	NA/ Soil	UFAM. C – Mao	100%	*Bacillus* sp MT052654
SX07	LB/ Water	Dona Chagas – Mao	100%	*Bacillus* sp MT052650
SPO7	LB/ Water	Portela farm – Mao	100%	*Bacillus* sp MT052638.1
SP1	LB/ Soil	Parananema - Pin	100%	*Bacillus* sp MT052642
SP6	LB/ Soil	Parananema - Pin	100%	*Bacillus* sp MT052653
SBC1	LB/ Soil	Science Grove – Mao	100%	*Bacillus* sp MT052667
SX05	NA/ Water	Dona Chagas – Mao	100%	*Bacillus* sp MT052644
SX01	NA/ Water	Dona Chagas – Mao	100%	*Bacillus* sp MT052646
SX02	NA/ Water	Dona Chagas – Mao	100%	*Bacillus* sp MT052611
SP8	NA/ Soil	Parananema - Pin	100%	*Bacillus* sp MT052659
SBC11	ISP2/ Soil	Science Grove – Mao	100%	*Bacillus* sp MT052660
SPO9	NA/ Water	Portela farm – Mao	100%	*Bacillus amyloliquefaciens* MT052630
SPa03	NA/ Soil	Parananema - Pin	100%	*Bacillus* sp MT052618
SX16	NA/ Water	Dona Chagas – Mao	100%	*Bacillus amyloliquefaciens* MT052665.1
SUF06	ISP2/ Soil	UFAM. C – Mao	100%	*Bacillus* sp MT052628
SBC3	LB/ Soil	Science Grove – Mao	100%	*Bacillus* sp MT052613
SPa12	LB/ Soil	Parananema - Pin	100%	*Bacillus* sp MT052612
AMc01	NA/ Water	M. River – Pin	100%	*Bacillus* sp MT052656
AP01	NA/ Water	Portela farm – Mao	100%	*Bacillus megaterium* MT052622
SX06	NA/ Water	Dona Chagas – Mao	100%	*Bacillus megaterium* MT052643
APO3	NA/ Water	Portela farm – Mao	100%	*Bacillus* sp MT052623
SPa08	NA/ Soil	Parananema - Pin	100%	*Bacillus* sp MT052610
SMP1.1	ISP2/ Soil	Macurany - Pin	100%	*Bacillus* sp MT052614
LFP1	NA/ Water	Fr. Pond – Pin	100%	*Bacillus* sp MT052619
SPO10	LB/ Water	Portela farm – Mao	100%	*Bacillus* sp MT052600
SMP1.2	ISP2/ Soil	Macurany - Pin	100%	*Bacillus* sp MT052635
SX13	LB/ Water	Dona Chagas – Mao	100%	*Bacillus* sp MT052640
SPa01	LB/ Soil	Parananema - Pin	100%	*Bacillus* sp MT052617
SUF.04	LB/ Soil	UFAM. C – Mao	100%	*Bacillus* sp MT052616
SUF05.1	LB/ Soil	UFAM. C – Mao	100%	*Bacillus* sp MT052664
SUF01	NA/ Soil	UFAM. C – Mao	100%	*Bacillus* sp MT052657
LFP3	ISP2/ Water	Fr. Pond – Pin	100%	*Bacillus* sp MT052655
SX10	ISP2/ Water	Dona Chagas – Mao	100%	*Bacillus* sp MT052663
SPa14	ISP2/ Soil	Parananema - Pin	100%	*Bacillus* sp MT052651
SBC9	ISP2/ Soil	Science Grove – Mao	100%	*Bacillus* sp MT052662
SBC10	ISP2/ Soil	Science Grove – Mao	100%	*Bacillus* sp MT052628
SBC12	NA/ Soil	Science Grove – Mao	100%	*Bacillus pumilus* MT052606
SPO2	LB/ Water	Portela farm – Mao	100%	*Bacillus safensis* MT052609
SX15	LB/ Water	Dona Chagas – Mao	100%	*Bacillus safensis* MT163316
SPO11	LB/ Water	Portela farm – Mao	100%	*Bacillus safensis* MT052625

SPa22	LB/ Soil	Parananema - Pin	100%	*Bacillus safensis* MT052639
SPa02	LB/ Water	Portela farm – Mao	100%	*Bacillus* sp MT052621
SBC2	LB/ Soil	Science Grove – Mao	100%	*Bacillus subtilis* MT052632
LFP2	LB/ Water	Fr. Pond – Pin	100%	*Bacillus subtilis* MT052629
SPa10	NA/ Soil	Parananema - Pin	100%	*Bacillus* sp MT052631
SC15.10	ISP2/ Soil	Science Grove – Mao	100%	*Bacillus subtilis* MT052615
SP12	ISP2/ Soil	Parananema - Pin	100%	*Bacillu* sp MT052603
SP06	LB/ Soil	Parananema - Pin	100%	*Bacillus* sp MT052669
SBC13	NA/ Soil	Science Grove – Mao	100%	*Bacillus* sp MT052634
SC15.7	NA/ Soil	H15. INPA – Mao	100%	*Bacillus* sp MT052605
SX04	NA/ Water	Dona Chagas – Mao	100%	*Bacillus* sp MT052645
GD02.13	NA/ Water	Gordo Farm - Coa	100%	*Bacillus* sp MT163315
SPa09	NA/ Soil	Parananema - Pin	100%	*Bacillus* sp MT052636
SMP1.3	ISP2/ Soil	Macurany - Pin	100%	*Bacillus* sp MT052648.1
SX08	LB/ Water	Dona Chagas – Mao	100%	*Bacillus velezensis* MT052649
SUF05	LB/ Soil	UFAM. C – Mao	100%	*Bacillus* sp MT052624
APR1.2	NA/ Soil	Pa. Stream – Pin	100%	*Bacillus velezensis* MT052627
APR1.1	NA/ Soil	Pa. Stream – Pin	100%	*Bacillus velezensis* MT052626
SPO5	LB/ Water	Portela farm – Mao	100%	*Bacillus* sp MT052608
SX13.1	LB/ Water	Dona Chagas – Mao	100%	*Bacillus* sp MT052668
Ara5	NA/ Water	Aerial Stream - Pin	100%	*Bacillus* sp MT052601
SPa06	NA/ Soil	Parananema - Pin	100%	*Bacillus* sp MT052607
SPO13	ISP2/ Water	Portela farm – Mao	100%	*Bacillus* sp MT052652
Apr14	ISP2/ Water	Parananema - Pin	100%	*Bacillus* sp MT052595
Apr6	ISP2/ Water	Parananema - Pin	100%	*Bacillus* sp MT052596
SP1	ISP2/ Soil	Parananema - Pin	100%	*Bacillus* sp MT052602
AMc06	ISP2/ Water	M. River – Pin	100%	*Bacillus zhangzhouensis* MT052666
SX17.1	NA/ Water	Dona Chagas – Mao	100%	*Bacillus zhangzhouensis* MT052641
SPO	ISP2/ Water	Portela farm – Mao	100%	*Bacillus zhangzhouensis* MT052661
SPa07	NA/ Soil	Parananema - Pin	100%	*Brevibacillus halotolerans* MT052647
SPa04	NA/ Soil	Parananema - Pin	100%	*Brevibacillus halotolerans* MT052633
SPa13	LB/ Soil	Parananema - Pin	100%	*Brevundimonas olei* MT052637
Apr8	ISP2/ Water	Pa. Stream – Pin	100%	*Klebsiella* sp MT052599
Apr9	ISP2/ Water	Pa. Stream – Pin	100%	*Serratia* sp MT052597
				77 Bacterian strains

The sequence identities were 100% similar to the NCBI Genbank sequences and Ribossomal Database Project (RDP-II).
Pin=Parintins, Mao=Manaus and Coa=Coari; UFAM. C – Mao=UFAM Campus I – Mao; M. River – Pin=Macurany River – Pin; Fr. Pond – Pin=Francesa Pond – Pin; H15. INPA – Mao=House 15 INPA – Mao; Pa. Stream – Pin=Parananema. Stream – Pin.

Table S2. (A-D) - LC_{50} and LC_{90} values of microbial cultures larvicidal activity against *Aedes aegypti* larvae. LC_{50} and LC_{90} were assessed by Probit, with p ≤ 0,05 [31], using the statistical software Polo Plus 1.0 (LeOra Software, CA. USA) [32]. Lethal concentrations and confidence interval (CI 95%) were analyzed by the Lilliefors normality test (K samples), analysis of variance (ANOVA), multiple comparison Tukey test (p ≤ 0,05) and Student's t test with the software BioEstat 5.3 for Windows [33].

Table S2. A - Supernatant (SUP) fractions.

**Strain**	**LC_{50} (CI 95%)**	**LC_{90} (CI 95%)**	X^2	*DF*	**Slope (±SE)**
24 h					

Bti BR101	0.009 (0.002 – 0.013) a	0.048 (0.027 – 0.813) a	5.4296	2	1.719 (±0.171)
SX15	0.006 (0.001 – 0.010) a	0.068 (0.037 – 0.855) a	9.0326	3	1.252 (±0.146)
SPa07	0.007 (0.002 – 0.010) a	0.056 (0.031 – 0.901) a	10.497	3	1.418 (±0.157)
48 h					
Bti BR101	0.008 (0.006 – 0.010) a	0.029 (0.022 – 0.053) a	6.7637	3	2.347 (±0.200)
SX15	0.006 (0.001 – 0.010) a	0.068 (0.037 – 0.855) a	9.0326	3	1.252 (±0.146)
SPa07	0.006 (0.002 – 0.009) a	0.020 (0.015 – 0.059) a	18.495	3	2.599 (±0.216)
72 h					
Bti BR101	0.004 (0.001 – 0.007) a	0.004 (0.001 – 0.007) a	7.4621	3	2.748 (±0.365)
SX15	0.005 (0.001 – 0.007) a	0.005 (0.001 – 0.007) a	6.7782	3	2.864 (±0.389)
SPa07	0.004 (0.001 – 0.007) a	0.004 (0.001 – 0.007) a	8.0917	3	2.687 (±0.355)

LC Lethal Concentration; CI = Confidence Interval; χ^2 = chi-square; DF = Degrees of freedom; SE = Standard Error. Same letters, a, b, c, etc do not differ at the 5% probability level ($p > 0.05$).

Table S2. B - Non-autoclaved Pellet (PEL) fractions.

**Strain**	**LC_{50} (CI 95%)**	**LC_{90} (CI 95%)**	***X^2***	***DF***	**Slope (±SE)**
24 h					
Bti BR101	0.008 (0.004 – 0.011) bc	0.058 (0.033 – 0.391) a	6.5449	3	1.500(±0.180)
SPa09	0.006 (0.001 – 0.009) c	0.089 (0.089 – 3.192) a	6.4172	3	1.072 (±0.152)
SPa22	0.013 (0.010 – 0.016) ab	0.113 (0.064 – 0.405) a	9.2917	4	1.369 (±0.127)
GD02.13	0.006 (0.002 – 0.009) c	0.148 (0.068 – 1.428) a	6.2864	4	2.391 (±0.340)
SP06	0.009 (0.006 – 0.012) bc	0.083 (0.050 – 0.266) a	9.3171	4	1.358 (±0.130)
SBC13	0.007 (0.004 – 0.009) c	0.009 (0.044 – 0.192) a	11.998	5	1.289 (±0.125)
SPa07	0.009 (0.004 – 0.013) bc	0.124 (0.0060 – 1.097) a	10.931	4	1.130 (±0.126)
SX15	0.010 (0.006 – 0.013) bc	0.088 (0.056 – 0.231) a	3.7187	3	1.348 (±0.125)
BC1	-	-	-	-	-
48 h					
Bti BR101	0.008 (0.007 – 0.009) a	0.023 (0.021 – 0.027) a	1.988	2	2.716 (±0.225)
SPa09	0.006 (0.002 – 0.009) ab	0.026 (0.019 – 0.055) a	3.5529	2	2.049 (±0.185)
SPa22	0.004 (0.001 – 0.007) ab	0.023 (0.018 – 0.042) a	7.2396	3	1.762 (±0.185)

GD02.13	0.005 (0.001 – 0.008) ab	0.017 (0.012 – 0.03) a	12.748	3	2.260 (±0.225)
SP06	0.007 (0.002 – 0.010) ab	0.029 (0.029 – 0.091) a	5.3146	2	2.052 (±0.175)
SBC13	0.004 (0.001 – 0.006) b	0.019 (0.015 – 0.026) a	4.8732	3	1.871 (±0.201)
SPa07	0.005 (0.001 – 0.008) ab	0.021 (0.016 – 0.0875) a	12.227	3	2.122 (±0.227)
SX15	0.005 (0.001 – 0.008) ab	0.022 (0.016 – 0.084) a	12.227	3	2.061 (±0.225)
BC1	-	-	-	-	-
72 h					
Bti BR101	0.003 (0.000 – 0.006) a	0.012 (0.009 – 0.016) a	30.173	6	2.337 (±0.284)
SPa09	0.005 (0.001 – 0.007) a	0.015 (0.012 – 0.021) a	8.4219	3	2.595 9±0.260)
SPa22	0.005 (0.001 – 0.007) a	0.015 (0.012 – 0.021) a	8.4219	3	2.595 (±0.260)
GD02.13	0.003 (0.000 – 0.005) a	0.012 (0.009 – 0.016) a	16.803	5	1.916 (±0.235)
SP06	0.007 (0.002 – 0.009) a	0.018 (0.014 – 0.046) a	6.0876	2	3.099 (±0.288)
SBC13	0.005 (0.003 – 0.006) a	0.012 (0.010 – 0.013) a	0.557	1	3.234 (±0.529)
SPa07	-	-	-	-	-
SX15	-	-	-	-	-
BC1	0.023 (0.021 – 0.027) a	0.256 (0.159 – 0.535) a	2.458	3	1.234 (±0.137)

LC Lethal Concentration; CI = Confidence Interval; χ^2 = chi-square; DF = Degrees of freedom; SE = Standard Error. Same letters, a,b,c, etc do not differ at the 5% probability level ($p > 0.05$).

Table S2. C - Autoclaved pellet (APEL) fractions.

Strain	LC_{50} (CI 95%)	LC_{90} (CI 95%)	X^2	*DF*	Slope (±SE)
24h					
SPa22	0.009 (0.003 – 0.013) a	0.073 (0.040 – 0.670) a	9.8656	3	1.389 (±0.145)
48h					
SPa22	0.006 (0.001 – 0.010) a	0.025 (0.017 – 0.106) a	6.7376	2	2.130 (±0.187)
72h					
SPa22	0.007 (0.004 – 0.009) a	0.018 (0.014 – 0.034) a	63.089	6	3.313 (±0.232)

LC Lethal Concentration; CI = Confidence Interval; χ^2 = chi-square; DF = Degrees of freedom; SE = Standard Error. Different letters differ at the 5% probability level ($p > 0.05$).

Table S2. D - Mixed autoclaved pellet plus supernatant (APEL+SUP) fractions.

Strain	LC_{50} (CI 95%)	LC_{90} (CI 95%)	X^2	*DF*	Slope (±SE)

24h					
Bti BR101	0.005 (0.002 – 0.008) a	0.037 (0.027 – 0.078) a	5.3019	3	1.495 (±0.160)
SPa22	0.007 (0.002 – 0.010) a	0.031 (0.021 – 0.115) a	33.418	3	2.030 (±0.155)
SP06	0.008 (0.004 – 0.011) a	0.046 (0.030 – 0,122) a	8.1382	3	1.680 (±0.143)
48h					
Bti BR101	0.005 (0.001 – 0.008) a	0.018 (0.013 – 0.039) a	28.230	4	2.252 (±0.204)
SPa22	0.007 (0.004 – 0.009) a	0.018 (0.014 – 0.032) a	59.140	6	3.337 (±0.234)
SP06	0.006 (0.002 – 0.009) a	0.023 (0.017 – 0.074) a	12.453	3	2.252 (±0.204)
72h					
Bti BR101	0.004 (0.000 – 0.006) a	0.011 (0.010 – 0.015) a	16.042	5	2.607 (±0.367)
SPa22	0.003 (0.000 – 0.006) a	0.012 (0.009 – 0.016) a	17.313	5	2.607 (±0.367)
SP06	0.003 (0.000 – 0.005) a	0.016 (0.013 – 0.040) a	26.194	6	1.776 (±0.239)

LC Lethal Concentration; CI = Confidence Interval; χ^2 = chi-square; DF = Degrees of freedom; SE = Standard Error. Different letters differ at the 5% probability level ($p > 0.05$).
